# Hypercobalaminemia Leading to the Diagnosis of Retroperitoneal Paraganglioma: A Case Report

**DOI:** 10.7759/cureus.101156

**Published:** 2026-01-09

**Authors:** Beatriz Vitó Madureira, Daniela Soares, Sara Vasconcelos, Sofia C. Barbosa, Sara Santos

**Affiliations:** 1 Internal Medicine, Local Health Unit of Entre Douro e Vouga, Santa Maria da Feira, PRT

**Keywords:** hypercobalaminemia, metanephrines, neuroendocrine tumors, paraganglioma, retroperitoneal mass

## Abstract

Pheochromocytomas and paragangliomas (PPGLs) are rare neuroendocrine tumors with heterogeneous clinical presentations, ranging from classic catecholamine-related symptoms to incidental imaging findings. Hypercobalaminemia is most commonly associated with hematologic disorders, liver disease, or solid malignancies, and has been described in association with neuroendocrine tumors; however, a specific association with PPGLs has not been previously reported. We describe the case of a 47-year-old normotensive woman referred for evaluation of persistently elevated serum vitamin B12 levels. Apart from intermittent abdominal discomfort, she was otherwise asymptomatic. After exclusion of common causes of hypercobalaminemia, abdominal imaging revealed a retroperitoneal para-aortic mass. Histopathological and immunohistochemical analyses were consistent with PPGLs, and biochemical testing demonstrated elevated plasma and urinary normetanephrine levels, supporting the diagnosis of a functioning paraganglioma. ^68^Ga-DOTATOC positron emission tomography (PET) confirmed somatostatin receptor expression without evidence of additional disease, and the patient underwent complete surgical resection.

This case illustrates an atypical diagnostic pathway in which isolated hypercobalaminemia prompted further investigation and led to the diagnosis of a retroperitoneal paraganglioma. It reinforces that PPGLs may occur without classic catecholamine-related symptoms and highlights the clinical relevance of unexplained hypercobalaminemia as a laboratory finding warranting further evaluation. Persistent elevation of serum vitamin B12 levels following tumor resection suggests an incidental finding or a possible association with another, as yet unidentified, neoplastic process, emphasizing the importance of continued follow-up.

## Introduction

Pheochromocytomas and paragangliomas (PPGLs) are rare neuroendocrine tumors derived from chromaffin cells of the adrenal medulla or extra-adrenal paraganglia. Their clinical presentation is highly variable, ranging from classic catecholamine-related symptoms to incidental findings on imaging studies [1]. Early diagnosis is essential, as untreated PPGLs are associated with significant cardiovascular morbidity and mortality [2,3].

Hypercobalaminemia, defined as elevated serum vitamin B12 levels, may reflect underlying hematologic disorders, liver or renal disease, or solid malignancies, often due to increased production of cobalamin-binding proteins by proliferating cells [4,5]. Although associations between hypercobalaminemia and neuroendocrine tumors have been described [6,7], a specific association with PPGLs has not been previously reported.

This case is presented to illustrate an atypical diagnostic pathway in which unexpected hypercobalaminemia prompted further investigation, ultimately leading to the diagnosis of a retroperitoneal paraganglioma in an otherwise asymptomatic, normotensive adult. It highlights the importance of expanding the differential diagnosis in the presence of unexplained laboratory abnormalities.

## Case presentation

A 47-year-old woman with no relevant past medical history, including no known hypertension, was referred to the internal medicine outpatient clinic for evaluation of persistently elevated vitamin B12 levels for one year. During evaluation, the patient reported intermittent abdominal discomfort and denied weight loss, fever, palpitations, diaphoresis, or changes in bowel habits. She also denied the use of vitamin B12-containing supplements or any family history of malignancy. Physical examination revealed a normotensive patient with a soft abdomen with mild tenderness on deep palpation but without palpable masses or organomegaly. No peripheral lymphadenopathy was identified.

Initial laboratory evaluation (Table 1) revealed mildly elevated serum vitamin B12 levels. Autoimmune screening was negative, tumor markers were within normal limits, and chronic liver and renal diseases were excluded.

**Table 1:** Laboratory findings during the diagnostic workup Laboratory results obtained during the evaluation of an asymptomatic patient with isolated hypercobalaminemia. Initial testing demonstrated mildly elevated serum vitamin B12 levels, with no other abnormal findings. Abnormal values are indicated by an asterisk (*). Reference ranges may vary according to laboratory standards.

Parameter		Result	Unit	Reference Range
Hemoglobin		14.3	g/dL	12.0 - 16.0
White blood cells		4.0	x10⁹/L	4.0 – 11.0
Platelets		207	x10⁹/L	150 - 450
Peripheral blood smear		Normal	-	-
International normalized ratio (INR)		1.1	-	-
Iron		167	µg/dL	50 - 170
Total iron-binding capacity (TIBC)		275	µg/dL	250 – 425
Transferrin saturation		45.8	%	15.0 – 50.0
Ferritin		183.08	ng/mL	4.63 – 204.00
Folic acid		8.4	ng/mL	3.0 – 20.0
B12 vitamin		1204*	pg/mL	189 – 883
Urea		25	mg/dL	15 - 40
Creatinine		0.7	mg/dL	0.6 – 1.1
Sodium		139	mmol/L	136.0 – 145.0
Potassium		4.4	mmol/L	3.5 – 5.1
Total bilirubin		0.55	mg/dL	0.20 – 1.20
Aspartate aminotransferase (AST)/Alanine aminotransferase (ALT)		14 / 40	U/L	5 - 34 / 0 - 55
Gamma-glutamyl transferase (GGT)/Alkaline phosphatase (ALP)		15 / 41	U/L	<38 / 40 - 150
Erythrocyte sedimentation rate		14	mm	0.0 – 20
C-reactive protein (CRP)		<1.0	mg/L	0.0 – 5.0
Antinuclear antibodies (ANA)		0.2	U/mL	Negative <0.7
				Positive >1.0
Myeloperoxidase anti-neutrophil cytoplasmic antibodies (MPO-ANCA)		0.20	Ul/mL	Negative <3.5
				Positive >5
Anti-proteinase 3 antibodies (PR3-ANCA)		0.30	Ul/mL	Negative <2
				Doubtful 2-3
				Positive >3
Rheumatoid factor		<20	Ul/mL	<30
Anti-cyclic citrullinated peptide (anti-CCP) antibodies		3.9	U/mL	<5.0
Alpha-fetoprotein		<2	ng/mL	<8
Β2 microglobulin		1.75	mg/L	<2.64
Protein electrophoresis		Normal	-	-
Thyroid-stimulating hormone (TSH)		1.56	µUl/mL	0.35 – 4.94
Free thyroxine (FT4)		11.2	pmol/L	9.0 – 19.0
Urinalysis		Negative for proteinuria, leukocyturia, and erythrocyturia	-	-

**Figure 1:**
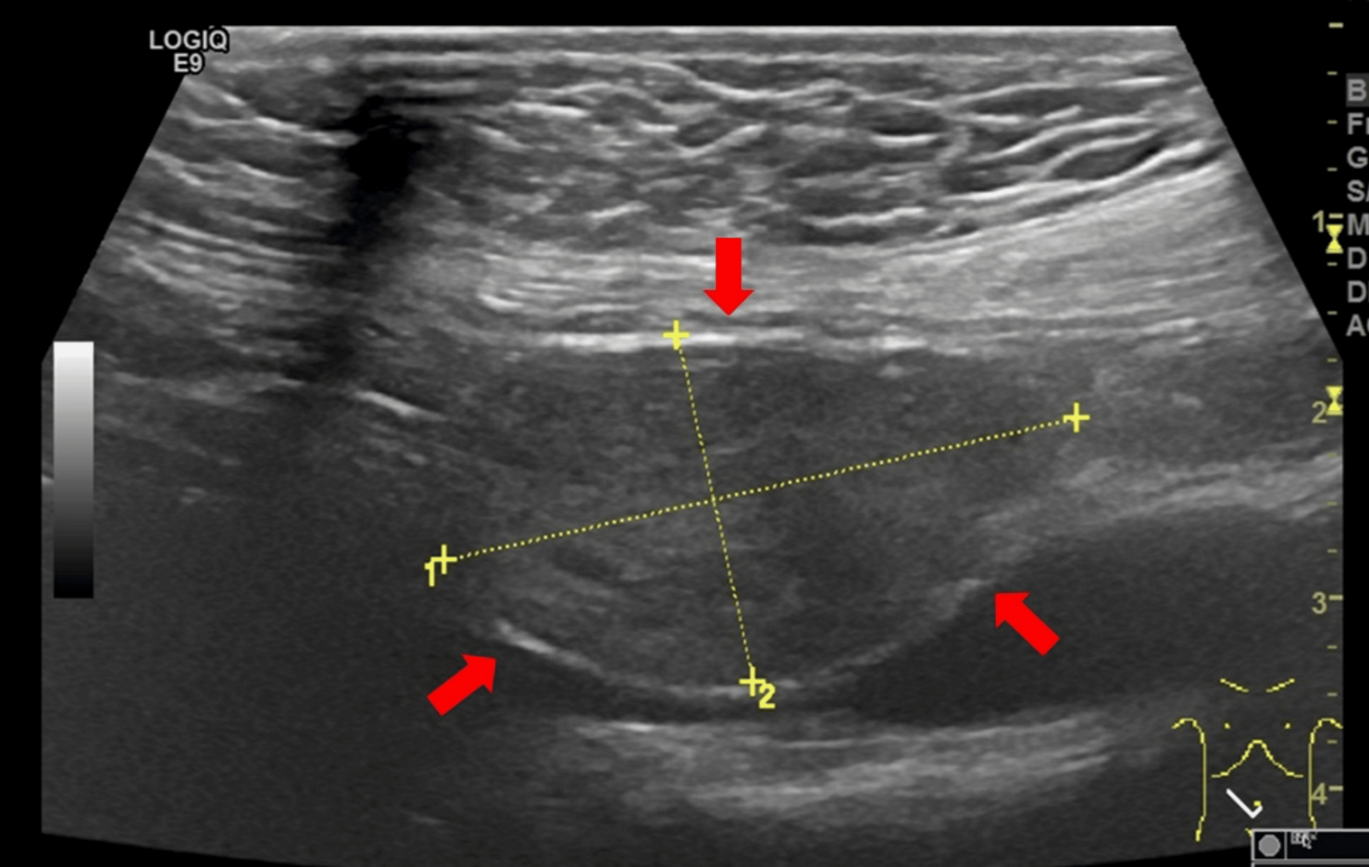
Abdominal ultrasound showing a retroperitoneal mass. Abdominal ultrasound showing a well-circumscribed hypoechoic solid lesion in the interaortocaval region of the retroperitoneum (red arrows), measuring approximately 24 mm in its greatest diameter, as indicated by the caliper measurements.

Abdominal ultrasound showed a 24mm hypoechoic nodular lesion in the supraumbilical region within the interaortocaval space (Figure 1). 

Given the indeterminate nature of this finding, a contrast-enhanced computed tomography (CT) scan was performed, which confirmed a 28-mm solid para-aortic mass in the right retroperitoneum, warranting further characterization. An ultrasound-guided biopsy of the lesion was performed, without documented post-procedural complications. Histopathological examination revealed a solid-pattern neoplasm composed of cells with abundant amphophilic cytoplasm and ovoid nuclei, occasionally displaying prominent nucleoli. Immunohistochemical analysis demonstrated preserved expression of GATA-binding protein 2 (GATA-2), synaptophysin, chromogranin, and succinate dehydrogenase complex iron-sulfur subunit B (SDHB). There was no expression of cytokeratins (CAM-5.2), paired box gene 8 (PAX-8), or SRY-related HMG-box gene 10 (SOX-10), and the Ki-67 proliferative index was 2%. These findings were consistent with a diagnosis of paraganglioma or pheochromocytoma.

Subsequent targeted biochemical evaluation revealed elevated plasma and 24-hour urinary normetanephrine levels (291.0 pg/mL and 1151.0 µg/24 h, respectively), while plasma and urinary catecholamine levels were within normal limits (Table 2), supporting the diagnosis of a retroperitoneal paraganglioma.

**Table 2:** Biochemical evaluation of catecholamine and metanephrine secretion Plasma and 24-hour urinary metanephrine, normetanephrine, and catecholamine levels were obtained during the diagnostic evaluation. Elevated serum and urinary normetanephrine levels are highlighted with an asterisk (*), while other parameters remained within reference ranges. Reference ranges are provided according to laboratory standards.

Parameter		Result	Unit	Reference Range
Metanephrines				
Metanephrine	Serum	46.0	pg/mL	<100.0
	Urinary	164.0	µg/24H	<341.0
Normetanephrine	Serum	291.0*	pg/mL	<216.0
	Urinary	1151.0*	µg/24H	<444.0
Catecholamines				
Adrenaline	Serum	<15	pg/mL	<60
	Urinary	8.0	µg/24H	<18
Norepinephrine	Serum	73	pg/mL	200 – 800
	Urinary	53	µg/24H	<76
Dopamine	Serum	<15	pg/mL	<150
	Urinary	272.0	µg/24H	<390

**Figure 2:**
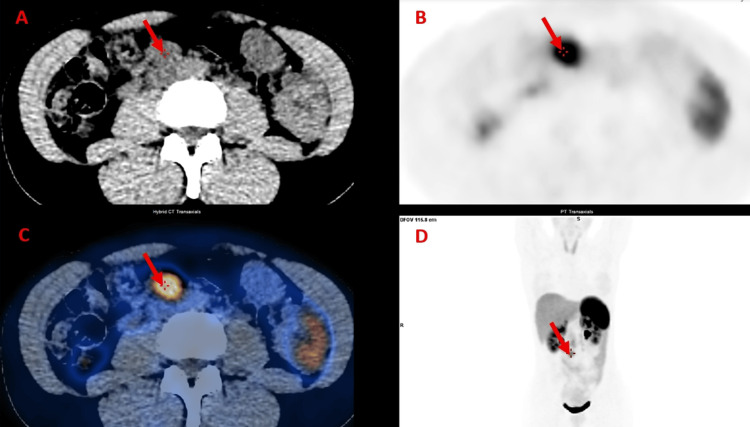
DOTATOC PET/CT demonstrating a hypermetabolic retroperitoneal nodule consistent with paraganglioma Axial CT (A), axial ^68^Ga-DOTATOC PET (B), fused ^68^Ga-DOTATOC PET/CT (C), and coronal ^68^Ga-DOTATOC PET (D) images show a solitary 25mm hypermetabolic retroperitoneal nodule (red arrow), located in the right para-aortic region. The lesion demonstrates intense radiotracer uptake consistent with somatostatin receptor overexpression, supporting the diagnosis of paraganglioma. No additional sites of abnormal uptake are identified.

A ^68^Ga-DOTATOC positron emission tomography (PET) scan confirmed the presence of a 25-mm hypermetabolic retroperitoneal nodule with somatostatin receptor overexpression (maximum standardized uptake value (SUVmax) 7.8), without evidence of additional lesions (Figure 2).

Following PET imaging, the patient was started on preoperative selective α1-adrenergic blockade with doxazosin. Complete surgical resection of the mass was performed one month later, without postoperative complications. At one-month follow-up, the patient remained asymptomatic with no evidence of disease recurrence, despite persistently mildly elevated serum vitamin B12 levels (998pg/mL).

## Discussion

PPGLs are rare neuroendocrine tumors and remain diagnostically challenging due to their heterogeneous clinical presentation [1]. Contemporary reviews estimate an incidence of approximately two to eight cases per million per year [3]. Up to one-third of cases are identified incidentally during imaging performed for unrelated reasons, particularly when catecholamine secretion is minimal or absent [1]. The present case is noteworthy because the patient did not exhibit hypertension or adrenergic crises, reinforcing that biochemical activity does not always correlate with clinical presentation.

Diagnosis relied on a multimodal approach integrating imaging, biochemical testing, and histopathological evaluation. Cross-sectional imaging with CT enabled anatomical characterization of the lesion, while ^68^Ga-DOTATOC PET confirmed somatostatin receptor overexpression, a typical feature of well-differentiated neuroendocrine tumors and a valuable tool for localization and staging [8,9]. Biochemical evaluation revealed elevated plasma and urinary normetanephrines with normal catecholamine levels, consistent with extra-adrenal paragangliomas and in line with current recommendations supporting metanephrines as the most sensitive first-line biomarkers for PPGLs [2,3].

Histopathological and immunohistochemical analyses were central to establishing the diagnosis. Expression of synaptophysin and chromogranin supported the neuroendocrine nature of the tumor, while preserved GATA-2 expression favored paraganglionic differentiation [10]. The absence of CAM-5.2, PAX-8, and SOX-10 expression helped exclude epithelial, renal, melanocytic, and peripheral nerve sheath neoplasms [11,12]. Preservation of SDHB expression suggested an intact SDH complex, making SDH-deficient paraganglioma less likely and potentially indicating a lower probability of association with hereditary syndromes, although genetic testing remains essential for definitive risk stratification [13,14]. The low Ki-67 proliferative index (2%) was consistent with an indolent tumor profile; however, malignant potential in PPGLs cannot be reliably predicted by histologic or proliferative features alone [10].

An unusual aspect of this case was the presence of unexplained hypercobalaminemia, which triggered further diagnostic evaluation. Elevated serum cobalamin levels are most commonly associated with myeloproliferative disorders, liver disease, and solid tumors, often due to increased production of vitamin B12-binding proteins such as haptocorrin (TCN1) or transcobalamin (TCN2) [4,5]. Although neuroendocrine tumors have been associated with altered vitamin B12 metabolism [6,7], large-scale gene expression analyses of PPGLs have not identified TCN1 or TCN2 as relevant or highly expressed transcripts, making tumor-derived vitamin B12 secretion unlikely in this context [15]. This suggests that the hypercobalaminemia observed in this case was incidental or potentially related to another, as yet undiagnosed, occult malignancy. The persistence of elevated serum vitamin B12 levels after complete tumor resection further supports this interpretation.

This case emphasizes two important clinical considerations. First, PPGLs may present without the classic triad of symptoms, reinforcing the need for a high index of suspicion even in asymptomatic and normotensive patients [1,3]. Second, isolated hypercobalaminemia warrants systematic evaluation for underlying neoplastic or systemic disease when common causes have been excluded [4-6]. Continued follow-up is therefore essential, including consideration of additional imaging modalities, such as fluorodeoxyglucose (FDG)-PET, to identify potential DOTATOC-negative malignancies, and genetic testing to assess for possible germline mutations [9].

## Conclusions

This case illustrates an atypical diagnostic pathway in which unexplained hypercobalaminemia served as the initial clue leading to the diagnosis of a retroperitoneal paraganglioma in an asymptomatic, normotensive patient. It reinforces the heterogeneous clinical presentation of PPGLs and the need to maintain a high index of suspicion even in the absence of classic symptoms. Additionally, this report highlights the clinical relevance of isolated hypercobalaminemia as a laboratory finding that warrants further investigation for underlying neoplastic processes when common causes have been excluded. The persistent elevation of serum vitamin B12 levels following tumor resection suggests that hypercobalaminemia may have been incidental or potentially related to another, as yet unidentified, occult malignancy. Ongoing follow-up is therefore essential to exclude alternative etiologies.
